# “Blue flags”, development of a short clinical questionnaire on work-related psychosocial risk factors - a validation study in primary care

**DOI:** 10.1186/s12891-017-1677-z

**Published:** 2017-07-24

**Authors:** Charlotte Post Sennehed, Gunvor Gard, Sara Holmberg, Kjerstin Stigmar, Malin Forsbrand, Birgitta Grahn

**Affiliations:** 1 Department of Clinical Sciences Lund, Lund University, Orthopedics, Lund, Sweden; 2 Epidemiology and Register Centre South, Region Skåne, Lund, Sweden; 3 Department of Research and Development, Region Kronoberg, Box 1223, 35112 Växjö, Sweden; 4 Department of Health Sciences, Lund University, Lund, Sweden; 5 Department of Health Sciences, Luleå University of Technology, Luleå, Sweden; 6 Division of Occupational and Environmental Medicine, Institute of Laboratory Medicine, Lund University, Lund, Sweden; 7 Blekinge Centre of Competence, Landstinget Blekinge, Karlskrona, Sweden

**Keywords:** Work-related risk factors, Psychosocial, Work ability, Questionnaire, Validity

## Abstract

**Background:**

Working conditions substantially influence health, work ability and sick leave. Useful instruments to help clinicians pay attention to working conditions are lacking in primary care (PC). The aim of this study was to test the validity of a short “Blue flags” questionnaire, which focuses on work-related psychosocial risk factors and any potential need for contacts and/or actions at the workplace.

**Methods:**

From the original“The General Nordic Questionnaire” (QPS_Nordic_) the research group identified five content areas with a total of 51 items which were considered to be most relevant focusing on work-related psychosocial risk factors. Fourteen items were selected from the identified QPS_Nordic_ content areas and organised in a short questionnaire “Blue flags”. These 14 items were validated towards the 51 QPS_Nordic_ items. Content validity was reviewed by a professional panel and a patient panel. Structural and concurrent validity were also tested within a randomised clinical trial.

**Results:**

The two panels (*n* = 111) considered the 14 psychosocial items to be relevant. A four-factor model was extracted with an explained variance of 25.2%, 14.9%, 10.9% and 8.3% respectively. All 14 items showed satisfactory loadings on all factors. Concerning concurrent validity the overall correlation was very strong r_s_ = 0.87 (*p* < 0.001).). Correlations were moderately strong for factor one, r_s_ = 0.62 (*p* < 0.001) and factor two, r_s_ = 0.74 (*p* < 0.001). Factor three and factor four were weaker, bur still fair and significant at r_s_ = 0.53 (*p* < 0.001) and r_s_ = 0.41 (*p* < 0.001) respectively. The internal consistency of the whole “Blue flags” was good with Cronbach’s alpha of 0.76.

**Conclusions:**

The content, structural and concurrent validity were satisfactory in this first step of development of the “Blue flags” questionnaire. In summary, the overall validity is considered acceptable. Testing in clinical contexts and in other patient populations is recommended to ensure predictive validity and usefulness.

## Background

Working conditions are of great importance and influence health, work ability and sick leave [1]. Some conditions at work can be changed or adjusted to the individual, but other conditions are more difficult to modify. Health care practitioners and employers working together with accommodation strategies has been shown to be effective to promote health and work ability [2]. In Sweden, the employer’s responsibility for the work environment and work organisations is quite far-reaching and is regulated in law (Work Environment Act). This includes the physical work environment, but also the psychosocial and organisational working conditions. This means that the employer is responsible for doing systematic risk assessments on a regular basis and also take actions based on this [3, 4]. Patients with work disability are often seen in primary care (PC) and one of the PCs´ assignments is to support recovery and improve work ability, and therefore methods to help clinicians´ address work-related factors are needed.

There is evidence that the individuals´ working conditions are of great importance for patients with neck/back pain [5] and patients with symptoms of mental disorders [6]. Frequent neck/back pain combined with stress is associated with a high risk for reduced work ability [7]. Health, work and sick leave are all interrelated and low level of adjustment latitude at work can be a risk factor for sick leave [8–10]. Several studies confirm that work stress [7, 11], social support [12], balance between demands, control and support [13–15] are important factors that have an impact on work ability. Furthermore, psychological factors are important for return to work (RTW) among long-term sick leave patients [16–18]. This includes inequality and bullying at work, which are also important factors affecting health [19–21].

However, there is a lack of methods and relevant short questionnaires in PC to help clinicians in the consultation to pay attention to work-related factors that might influence the patient’s symptoms, diagnoses and potential for recovery. It may be appropriate, in addition to medical measures to advice patients to contact their employer about possible workplace adjustments or to ensure that occupational health services are engaged.

Screening for different health status or risks is common in health care in general and are often described as different type of clinical “flags”. The flag system has been developed for the assessment of risk factors and recommended as an investigative methodology and until now especially so in regards to musculoskeletal disorders (MSD) [22]. The identification of red and yellow flags is established and provides valuable information to clinicians in health care. Red flags are screening for severe health problems or diseases in need for more extensive diagnostic investigations [23] and yellow flags assess mental and emotional health risk factors [24].

Blue flags are defined as the individuals’ perceptions of work-related factors that can have an impact on disability. Screening for blue flags is intended for identification of work-related psychosocial risk factors, for example job dissatisfaction and/or poor colleague or supervisor relationships [25]. Earlier research indicates that health care should use questionnaires that cover these types of risk factors in order to support work ability [25, 26]. Work support [27] and formalised peer support at the workplace [28] has been found to be associated with reduced low back pain and reduction in sick leave. For this reason, there are recommendations that the examination of the patient also should include assessment of work-related psychosocial risk factors, which can predict the risk of chronic disabling back pain [29, 30]. The “Readiness for Return to Work scale” was developed to address the motivational factors contributing to RTW for workers with MSD on sick leave. The instrument is recommended to be used in planning and evaluation of occupational intervention/occupational rehabilitation [31]. Other questionnaires focusing blue flags, such as the Back Disability Risk Questionnaire (BDRQ) [32], the Occupational Role Questionnaire (ORQ) [33], the Obstacles to Return to Work Questionnaire (ORTWQ) [34] and the Psychosocial Aspects of Work Questionnaire (PAWQ) [35] are all designed to be used in occupational health settings, hospitals and rehabilitation clinics. They are not designed to be used for screening for work-related psychosocial risk factors among patients in PC.

Clinical work and patient assessment is different in PC as compared to occupational rehabilitation settings. The time available for each consultation is generally much shorter and the patient population is unselected. Many patients are in early stages of illness or disease when consulting PC for advice and medical evaluation of symptoms. The assorting function in PC is important and an approach that identifies disease, guides treatment, and prevents unnecessary medicalization is warranted. The importance of robust early screening methods helping clinicians to deliver relevant counselling and treatment is thus central in healthcare development and procedures [36–40]. Until now there is to our knowledge no useful instrument, that is easy to handle and that takes a short time to complete recommended to help professionals in PC to identify important work-related psychosocial risk factors that can affect health and work ability [26]. Thus, there is a need for a generic instrument designed for use in PC to identify and highlight psychosocial risk factors for work disability, which indicates the need of early contacts and/or actions at the workplace in addition to the medical efforts at the PC. This instrument is intended to be used by different professionals when meeting patients in working age who are at risk of sick leave.

“The General Nordic Questionnaire for Psychological and Social Factors at Work” (QPS_Nordic_) is an established well-known questionnaire for the assessment of psychological, social and organisational working conditions as well as individual work-related attitudes. QPS_Nordic_ is the most comprehensive, reliable and valid questionnaire used in the Nordic countries today. This questionnaire has been used for organisational development, documentation of changes in working conditions, evaluation of organisational interventions and research [41–48]. The questionnaire includes 129 items divided into 13 different content areas classified according to task level, social and organisational level and individual level [49]. QPS_Nordic_ was constructed after extensive development and published in 2000. Two data sets were collected in Sweden, Norway, Denmark and Finland within various occupational fields. The factor structure of the questionnaire and the structural of the scales was studied in the first data set (*n* = 1015). The second data set (*n* = 995) was used to test the structural and predictive validity of the scales. The internal consistencies (alpha values 0.60–0.88) and test-retest reliabilities (0.55–0.82) were studied for each scale. In the content areas concerning working conditions Cronbach’s alpha has been found to be 0.69–0.85 [49].

However, a clinical questionnaire in PC needs to be short and easy to handle and QPS_Nordic_ is too extensive to be useful in clinical practice. The aim of this study was to test the validity of a short “Blue flags” questionnaire, which focuses on work-related psychosocial risk factors and any potential need for contacts and/or actions at the workplace.

## Methods

### **Design**

This is a methodological study with focus on content, structural and concurrent validity. We conducted the study with two different populations; one for the content validity and a different population for the structural and concurrent validity.

### Instrument development

A short questionnaire, “Blue flags”, intended for use in PC is under development. In this first step we have focused on work-related psychosocial risk factors based on items from the major QPS_Nordic_. Our ambition was to limit the number of items in the new short questionnaire. The selection of items from the original QPS_Nordic_ was based on relevant scientific literature studies, clinical experience and competence in the research group. From the 13 established content areas in the original QPS_Nordic_ the research group identified five content areas with a total of 51 items which were considered to be most relevant when focusing on work-related psychosocial risk factors [5, 6, 50–53]. These areas were; job demands [41–43], social interactions [45, 47, 48], quantitative demands [44], equality [54, 55], bullying and harassment [46, 56]. Therefore the selected QPS_Nordic_ items covered these content areas with the following number of items; job demands (32 items), social interactions (6 items), quantitative demands (9 items), equality (2 items) and bullying and harassment (2 items). The answers in the QPS_Nordic_ are given on a 5 - point Likert scale from one to five (1 = no problems and 5 = most problems). Fourteen items were selected from the identified QPS_Nordic_ content areas and organized in a short questionnaire (“Blue flags”). This method is previous described as relevant in research when a long questionnaire is condensed into a shorter [57, 58]. The 14 items in the “Blue flags” questionnaire were 7 items on job demands, 2 items on social interactions, 2 items on quantitative demands, 2 items on equality and 1 item on bullying and harassment. The items related to equality and bullying have to some extent been reformulated to be better integrated in the “Blue flags”. The answers are given on a 5 - point Likert scale, as in the QPS_Nordic._

### Study populations and procedure

#### Content validity

One panel of professionals and one panel of patients were questioned in order to receive constructive feedback about the new short questionnaire [59–61]. Our intention was to have a broad and relevant representation of experience; both from pain rehabilitation, vocational rehabilitation and from PC. The intention was to gather information on the representativeness and clarity of the items by the panels´ constructive feedback as well as suggestions for improvement [62]. The recruitment criterion of the professional panel in health care was experience of work-related health issues. The recruitment criterion of the patient panel was their individual experience as a patient in PC with an episode of back pain and having risk for developing work disability. We were interested in their understanding of the items, perceived relevance and formulations. The panels were recruited from thirteen primary care centres (PCC), two occupational health services, one specialized pain rehabilitation centre and one inpatient centre in the southern parts of Sweden.

##### Professional panel

Sixty-five professionals from six units agreed to evaluate the short questionnaire “Blue flags” (19 men, 45 women) mean age 45 years (range 21–63 years). The represented professions were physiotherapists (*n* = 30), occupational therapists (*n* = 13), physicians (*n* = 8), social workers (*n* = 4), nurses (*n* = 6) and psychologists (*n* = 4). The professionals were working in health care, mostly in PC (65%) and occupational health (23%) and had been in health care for many years (74% ≥ 10 years). Information about the study was given through presentations at staff meetings and as written information. Professionals in the panel were asked to reflect on the relevance of the14 items when assessing the working conditions. They individually and anonymously evaluated the relevance of each item on a scale from one to three; 1 = not relevant, 2 = relevant and 3 = very relevant. They were also asked if there were items missing, unnecessary items or any need to rephrase items.

##### Patient panel

Consecutive patients at 13 PCCs were asked by physiotherapists to evaluate the 14 psychosocial items in the questionnaire “Blue flags”. Information about the study was given as written information. Forty-six patients from nine PCCs agreed to evaluate the items (10 men, 36 women), mean age 45 years (range 21–62 years), with pain problems in neck (*n* = 19), back/lumbar back (*n* = 24) and shoulder (*n* = 3). Patients were asked to consider whether the items could be helpful in an assessment regarding their working conditions. They individually and anonymously evaluated the relevance of each item on a scale from one to three; 1 = not relevant, 2 = relevant and 3 = very relevant. They were also asked if there were items missing, unnecessary items or any need to rephrase items.

#### Structural and concurrent validity

**Fig. 1:**
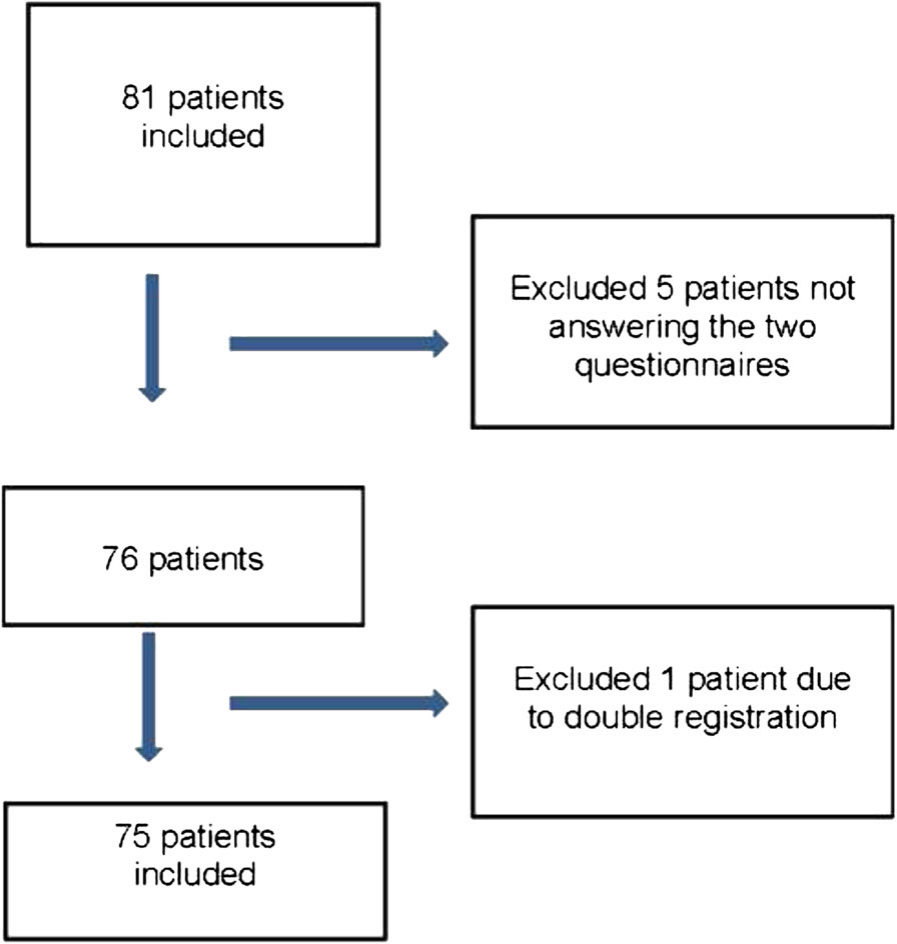
Flow chart, inclusion concurrent validity

To assess structural and concurrent validity a cohort of patients from a randomised clinical trial (WorkUp, ClinicalTrials.gov, ID NCT 02609750) answered both the short “Blue flags” questionnaire (14 items) and the original QPS _Nordic_ (51 items) during one visit to one of ten PCCs in southern Sweden. The patients were recruited consecutively in WorkUp when they applied for physiotherapy due to an episode of acute or subacute non-specific back pain and were identified as having risk for developing work disability according to the Örebro Musculoskeletal Pain Screening Questionnaire (ÖMPSQ), short form [57]. Other inclusion criteria in the WorkUp study were to not be currently on sick leave or being sickness absent less than 60 days. In all, 75 patients were included (73 with employment). Mean age was 44 years, (range 22–64 years). The PC patients completed the short “Blue flags” and the 51 corresponding items from the QPS_Nordic_ questionnaire during the visit to the physiotherapist. The patients also answered questions regarding their professional background (Fig. 1, Table 1).

**Table 1:** Descriptive data of the study population in the cross-sectional clinical study for concurrent validity, *n* = 75

	*n*	%
Women/men	50/25	67/33
Employment, yes	73	97.3
Profession		
Health care professions	24	32
Administration	18	24
Industrial/heavy-duty work	24	32
Education/service work	9	12
Type of employment		
Permanent	63	83
Temporary	4	5.3
Hourly	4	5.3
Missing	4	5.3
Time in current profession		
< 6 months	4	5
6–12 months	3	4
1–5 years	23	31
> 5 years	45	60
Sick-leave, yes	26	34.7

### Statistics

Data from questionnaires were manually entered in the database. SPSS 23.0 was used for all analysis.

#### Content validity

To compare the answers from the professional panel and the patient panel the ratings were dichotomised as relevant (relevant and very relevant) or not relevant. Due to small sample size or no answers Fishers Exact Test was used, two sided, to test the difference in proportions. *P*-values less than 0.05 were considered significant. The Content Validity Index (CVI) was used to test content validity [63]. We considered the items in “Blue flags” to be relevant if the item-level CVI was >78% per item. The overall “Blue Flags” was considered relevant if the average of the sum of CVI for each item for the entire scale was ≥90%.

#### Structural validity

An assessment of the factorability of the data was performed using Barlett’s test of sphericity and the Kaiser-Meyer-Olkin (KMO) measure of sampling adequacy [64]. Barlett’s test should be significant (*p* < 0.05) for the factor analysis to be considered appropriate. The KMO index ranges from 0 to 1, with 0.6 as a minimum value for a good factor analysis [64]. To investigate the factor structure of the “Blue flags” a factor analysis was performed using the principal components analysis (PCA) extraction with the Varimax rotation. A minimum eigenvalue of 1 was specified as extraction criterion and the criterion for factor loading was set at ≥0.5.

#### Concurrent validity

Concurrent validity was studied as the correlation between the 14 work-related psychosocial items in the “Blue flags” compared to the 51 corresponding items from the QPS_Nordic_ questionnaire. The items in both questionnaires have the same direction, i.e. a low value indicates better working conditions and answers that indicate problems have a higher value. Since both questionnaires provided ordinal data, we used a non-parametric approach and calculated Spearman’s rank correlation coefficient (r_s_) [65] between the two questionnaires. We had in accordance to Chan [66] set the limit in this study for values of r_s_ at 0.3–0.5 as fair correlation, r_s_ at 0.6–0.8 as moderately strong correlation and a very strong correlation at r_s_ > 0.8. Internal consistency was analysed by Cronbach’s alpha coefficient. We considered values of α ≥ 0.7 as good [67, 68].

### Ethics

The study was reviewed by the Regional Ethical Review Board in Lund, Sweden (Dnr 2012/497, 2013/426) and was approved 2013–06-12.

## Results

The 14 items on work-related psychosocial risk factors which were included in the “Blue flags” are shown in Table 2.

**Table 2:** The 14 work-related psychosocial risk factor items selected from the original QPS_Nordic_

Selected questions to the new short questionnaire “Blue flags”	Content areas
1. There are clear goals for my work	Job demands
2. My work contains positive challenges	QPS_Nordic_ 32 items
3. There are incompatible demands for me at my work	
4. I have control in my work situation	
5. I can solve problems that arise at work	
6. The work requires me to concentrate all the time and can make decisions	
7. My tasks at work are too difficult	
8. I can count on that if necessary, get help and support from my immediate supervisor	Social interactions
9. I can count on that if necessary, get help and support from my colleagues	QPS_Nordic_ 6 items
10. I can decide how fast I work	Quantitative demands
11. I have too many tasks, too much work to do	QPS_Nordic_ 9 items
12. Men and women are treated equally at my workplace	Equality
13. Old and young staff are treated equally at my workplace	QPS_Nordic_ 2 items
14. There has been bullying and harassment at my workplace during the last 6 months	Bullying and harassment QPS_Nordic_ 2 items

### Content validity

The two panels (*n* = 111) regarded the overall “Blue flags” items to be relevant, with a CVI of 90%. The range of the item level, CVI, was 73% - 97% (Table 3). A majority of the professionals considered each of the 14 psychosocial items in the “Blue flags” to be relevant. The patients were most doubtful when it came to “My tasks at work are too difficult” (41%) and “There has been bullying and harassment at my workplace during the last 6 months” (57%) (Table 3). The Fishers Exact Test showed significant differences in the distribution of the responses in the panels´ for nine items (Table 3). Twenty-three professionals and one patient gave suggestions about additional psychosocial items. In particular, they thought there could have been items concerning wellbeing at work (*n* = 20). Nineteen professionals and one patient gave a total of 40 suggestions about rephrasing items, especially concerning “There are clear goals for my work”, “There are incompatible demands for me at work”, “I have control in my work situation”, “I can solve problems that arise at work” and “I have too many tasks, too much work to do”. The item “My tasks at work are too difficult” was proposed to have space for comments.

**Table 3:** Distribution of the panels´ answers regarding the work-related psychosocial risk factor items in the “Blue flags”

	Total, *n* = 111				Professionals, *n* = 65				Patients, *n* = 46				p***
	Not relevant		Relevant/very relevant		Not relevant		Relevant/very relevant		Not relevant		Relevant/very relevant		
	*n*	%	*n*	%	*n*	%	*n*	%	*n*	%	*n*	%	
1. There are clear goals for my work^a^	8	7	101	93	2	3	62	97	6	13	39	87	0.063
2. My work contains positive challenges^a^	5	5	104	95	0	0	64	100	5	11	40	89	0.010
3. There are incompatible demands for me at my work^b^	9	8	97	92	2	3	60	97	7	16	37	84	0.032
4. I have control in my work situation^a^	3	3	107	97	0	0	64	100	3	6	43	94	0.070
5. I can solve problems that arise at work^a^	4	4	105	96	0	0	64	100	4	9	43	91	0.027
6. The work requires me to concentrate all the time and can make decisions^a^	12	11	97	89	3	5	60	95	9	20	37	80	0.027
7. My tasks at work are too difficult^a^	25	23	85	77	6	9	58	91	19	41	27	59	0.000
8. I can count on that if necessary, get help and support from my immediate supervisor	4	4	107	96	0	0	65	100	4	9	42	91	0.027
9. I can count on that if necessary, get help and support from my colleagues	3	3	108	97	0	0	65	100	3	7	43	93	0.068
10. I can decide how fast I work^a^	13	12	95	88	3	5	59	95	10	22	36	78	0.014
11. I have too many tasks, too much work to do^a^	11	10	98	90	1	2	64	98	10	23	34	77	0.000
12. Men and women are treated equally at my workplace^a^	13	12	95	88	5	8	58	92	8	18	37	82	0.142
13. Old and young staff are treated equally at my workplace^a^	18	16	91	84	9	14	54	86	9	20	37	80	0.602
14. There has been bullying and harassment at my workplace during the last 6 months	30	27	81	73	4	6	61	94	26	57	20	43	0.000

Missing data: ^a^missing ≤ 3, ^b^missing 5

***Fisher´s Exact Test, the relationship between the distribution of the responses for the professionals and the patients, significance if *p* < 0.05

### Structural validity

The suitability of the data for factor analysis was satisfactory with the KMO value of 0.6 and the Bartlett’s test with the significance of *p* < 0.001. All 14 items in the “Blue flags” showed satisfactory loadings with a range of 0.514–0.872. A four-factor model was extracted with a total variance explained of 59.4%. Each of the four factors explained 25.2%, 14.9%, 10.9% and 8.3% of the variance respectively. Factor one and two reflected two different aspects of job demands, namely job tasks and job control. Factor three reflected equality and factor four was mixed (Table 4).

**Table 4:** Factor analyses of the “Blue flags” (*n* = 75)

Rotated Component Matrix^a^				
	Factor 1	Factor 2	Factor 3	Factor 4
My tasks at work are too difficult	0.844			
There are incompatible demands for me at my work	0.713			
I have too many tasks, too much work to do	0.671			
I can count on that if necessary, get help and support from my colleagues	0.514			
I can count on that if necessary, get help and support from my immediate supervisor		0.723		
My work contains positive challenges		0.705		
There are clear goals for my work		0.637		
I can decide how fast I work		0.544		
I have control in my work situation		0.523		
Old and young staff are treated equally at my workplace			0.872	
Men and women are treated equally at my workplace			0.849	
I can solve problems that arise at work				0.614
There has been bullying and harassment at my workplace during the last 6 months				0.600
The work requires me to concentrate all the time and can make decisions				−0.569
Variance explained, initial eigenvalues (%)	25.2	14.9	10.9	8.3
Rotated sums of squared loadings,variance explained (%)	18.0	17.1	13.8	10.5

Extraction method: Principal Component Analysis. Rotation Method: Varimax with Kaiser Normalization

^a^Rotation converged in 7 iterations, rotation varimax

### Concurrent validity

Correlation between the 14 psychosocial items in “Blue flags” and the 51 corresponding items in QPS_Nordic_ showed very strong correlation, r_s_ = 0.87 (*p* < 0.001). Correlations between the “Blue flags” groups of items in the four factors and the corresponding QPS_Nordic_ items were moderately strong for factor one, r_s_ = 0.62 (*p* < 0.001), and factor two, r_s_ = 0.74 (*p* < 0.001). Factor three and factor four were weaker, but still fair and significant at r_s_ = 0.53 (*p* < 0.001) and r_s_ = 0.41 (*p* < 0.001) respectively (Table 5). The internal consistency of the whole “Blue flags” was good with Cronbach’s alpha of 0.76.

**Table 5:** Correlations between the “Blue flags” and QPS_Nordic_ using Spearman correlation coefficient^a^

	Blue Flags 14 items					
QPSNordic 51 items	Factor 1 Job demands, 4 items	Factor 2 Job tasks, 5 items	Factor 3 Equality, 2 items	Factor 4 Mixed, 3 items	All 14 items	*p* value^b^
Corresponding 20 items	0.62					< 0.001
Corresponding 18 items		0.74				< 0.001
Corresponding 2 items			0.53			< 0.001
Corresponding 11 items				0.41		< 0.001
All, 51 items					**0.87**	< 0.001

^a^Spearman’s rank correlation coefficient, r_s_ ≥ 0.6 was considered to indicate satisfying correlation

^b^ *p*-values <0.05 were considered significant

## Discussion

This manuscript presents the first preliminary development of a short clinical PC questionnaire focusing on work-related psychosocial risk factors. The “Blue flags” is intended to screen for such risk factors, and to identify any potential need for action at the workplace in addition to the medical interventions in PC. At this stage we denote the “Blue flags” as a questionnaire, but after further development the intention is a short, practical and useful screening tool for clinical practice. Recommendations have been made suggesting the use of screening methods in health care to identify patients in early stages with the purpose to guide them to the best treatment and avoid over-treatment [37–40]. Despite these recommendations, assessing work-related psychosocial risk factors and any potential need for contacts and/or actions at the workplace as a standardised procedure in PC is still not sufficiently established.

The study found satisfactory content validity, structural validity and concurrent validity for the new “Blue flags” questionnaire. The overall correlation for the work-related psychosocial risk factor items between the two questionnaires was very strong and for the factors it was fair to moderately strong. The professional panel and the patient panel had somewhat different views on the relevance of the items, where the professional panel assessed most of the items to be relevant, whereas two of the items were assessed as not relevant by 41–57% in the patient panel.

Regarding ten work-related psychosocial items more than 80% of the patients assessed the items to be relevant. There were differing opinions between professionals and patients especially when it came to the items “My tasks at work are too difficult” and “There has been bullying and harassment at my workplace during the last six months”. The patient panel had their own individual experience of being patients, unlike the professional panel who worked in the field. The majority of the professionals were highly educated in this area and had long experience of work in health care, on average more than 10 years. Most of them had experience concerning the relationship between work-related risk factors and health and generally they rated the relevance of the items higher than the patients. The patient panel responded to what they thought of the items in regards to assessing their own working conditions. The patient panel applied for physiotherapy treatment due to neck, back or shoulder pain and it might have been difficult to understand the items relevance in relation to their pain or in relation to their working conditions. Unfortunately we had no information as to whether their pain were related to their work, what type of jobs they had or even if they were currently employed. The level of satisfactory content validity was obtained regards the overall items with an average CVI of 0.9. However, the range of the items was broad (0.73–0.97) and this must be considered in regard to the two items mentioned above. Still, considering current research in the area of work-related psychosocial risk factors, we believe that items related to bullying and harassment [21, 69, 70] and job demands [71, 72] should be included in the questionnaire.

One third in the professional panel stated that there was a lack of items concerning wellbeing at work, for example relationships, conflicts and meaningfulness in the “Blue flags”. It is well known that wellbeing at work is an important psychosocial work area and an important aspect of the psychosocial environment [1, 13, 14]. Still, it is evident that all items in “Blue flags” are important components to summarise wellbeing at work and it is debatable if there is a need for additional items. We also have to consider rephrasing the items that the panel assessed to be unclear in the further development of the “Blue flags”.

In the first step the items were grouped in four content areas and one single item (bullying/harassment). This differed from the PCA distribution, where a four-factor solution was revealed, where bullying/harassment was included in the fourth mixed factor. These findings support the “Blue flags” as a whole questionnaire and as suitable for further development. The PCA result showed good loadings for all items. The factor structure supports our aim for further in-depth research in this area.

The correlation for the 14 psychosocial items in the new questionnaire with the 51 corresponding psychosocial items in QPS_Nordic_ was very strong. We had in accordance to Chan [66] defined values of r_s_ at 0.6–0.8 as moderately strong correlation and a very strong correlation at >0.8, which is a stricter definition than other studies [58, 73]. The correlation was considered good which indicates that the shorter “Blue flags” captured the work-related psychosocial items just as good as the longer questionnaire QPS_Nordic_ with 51 items. Both “Blue flags” and QPS_Nordic_ showed satisfactory internal consistency [67]. This is in line with previous evaluation of QPS_Nordic_ [49] and indicates that the 14 psychosocial items in the “Blue flags” is acceptable when it comes to internal consistency.

### Strengths and limitations

The intention was to develop a questionnaire for screening in PC and to guide clinicians towards the best action and treatment, including possible contacts and/or actions at the work place. This study did not include the establishment of cut-off points or analysis of predictive validity, which could be considered as limitations. Therefore, this questionnaire needs further development before it can be implemented in clinical practice.

To reduce the number of items and to ensure the construction of a comprehensive questionnaire we based our decisions on our clinical experience and recent research findings [41–47] so that the most important and relevant work-related psychosocial items in the original version were covered in the new short version. The QPS_Nordic_ items were tested in previous research [49] and the method of selecting items from the original long questionnaire to a short form is an established method [57, 58]. The extensive clinical and scientific experience from PC, occupational health, occupational rehabilitation and various professions (physician, physiotherapist and psychologist) strengthened the process when we condensed the number of items to the short “Blue flags”. The factor analysis confirmed that the items in this short version can be used as a stand-alone questionnaire.

When studying structural and concurrent validity, we included patients in the WorkUp study with no long-term work disability, although they were at risk for developing long-standing problems. It could also be a limitation since the study included only patients with acute and subacute pain in physiotherapy practice even though it is known that it is important to identify patients with work-related disabilities at an early stage [50–52]. Further studies could examine if it is possible to select patients to promote health and work ability and whether the “Blue flags” can indicate the need for early workplace actions. We also set higher level for concurrent validity compared to previous studies [58, 73], which is a strength. The “Blue flags” indicated satisfactory structural validity and internal consistency and this strengthen the results [67, 68].

The two different groups with patients who assessed either content validity (*n* = 46) or structural and concurrent validity (*n* = 75) were recruited from several PCCs and from different areas in southern Sweden, which strengthens the possibilities to generalize the results. The professional panel evaluating the content validity was chosen through personal contact and were not randomly selected. Despite this the range was broad concerning professions and they had extensive experience, which strengthens their trustworthiness. It could also be regarded as strength that there were two different groups of patients in the content and structural/concurrent analyses, respectively.

The result concerning content validity showed the relevance of the items and the importance of identifying work-related risk factors in PC. Furthermore, there were proposals for supplementary items in the questionnaire. The clinical utility needs to be further evaluated. There is also a need to test the questionnaire in other clinical contexts as well as in other patient contexts, such as those with long-standing MSD as well as those with mental disorders [74]. This Swedish questionnaire was tested in a Swedish context and future versions should therefore be validated in other languages and countries. A further step in the development of the “Blue flags” questionnaire could be to supplement it with other types of work-related risk factors that can influence work ability, such as ergonomic items. To examine the usefulness in clinical practice “Blue flags” needs to undergo further evaluation regarding feasibility and predictive validity for identification of the need of workplace interventions.

## Conclusions

The content, structural and concurrent validity were satisfactory in this first step of development of the “Blue flags” questionnaire. In summary, the overall validity is considered acceptable. Testing in clinical contexts and in other patient populations is recommended to ensure predictive validity and usefulness.

## Abbreviations

CVI: Content validity index
KMO: Kaiser-meyer-olkin measure
MSD: Musculoskeletal disorder
ÖMPSQ: The orebro musculoskeletal pain screening questionnaire
PC: Primary care
PCA: Principal components analysis
PCC: Primary care centre
QPS_Nordic_: The general nordic questionnaire for psychological and social factors at work
RCT: Randomised clinical trial
RTW: Return to work

## References

[1] Shain M, Kramer DM (2004). Health promotion in the workplace: framing the concept; reviewing the evidence. Occup Environ Med.

[2] Carroll C, Rick J, Pilgrim H, Cameron J, Hillage J (2010). Workplace involvement improves return to work rates among employees with back pain on long-term sick leave: a systematic review of the effectiveness and cost-effectiveness of interventions. Disabil Rehabil.

[3] Organisational and social work environment provisions, AFS 2015:4. Swedish Work Environment Authority; 2015. https://www.av.se/globalassets/filer/publikationer/foreskrifter/organisatorisk-och-social-arbetsmiljo-foreskrifter-afs2015_4.pdf. Accessed 8 June 2016.

[4] Organisational and social work environment provisions, AFS 2012:2. https://www.av.se/arbetsmiljoarbete-och-inspektioner/publikationer/foreskrifter/belastningsergonomi-afs-20122-foreskrifter/ Swedish Work Environment Authority; 2012 [Accessed 17 Nov 2016].

[5] Swedish Council on Health Technology Assessment S. Occupational exposures and back disorders (in Swedish). 2014. http://www.sbu.se/en/publications/sbu-assesses/occupational-exposures-and-back-disorders/. Accessed 1 Dec 2016: Contract No.: SBU report no 227.

[6] Swedish Council on Health Technology Assessment S. Occupational exposures and symptoms of depression and burnout (in Swedish), 2014. http://www.sbu.se/en/publications/sbu-assesses/role-of-the-work-environment-in-the-development-of-symptoms-of-depression-and-burnout/. Accessed 1 Dec 2016: Contract No.: SBU report no 223. 26803856

[7] Lindegard A, Larsman P, Hadzibajramovic E, Ahlborg G (2014). The influence of perceived stress and musculoskeletal pain on work performance and work ability in Swedish health care workers. Int Arch Occup Environ Health.

[8] Hultin H, Hallqvist J, Alexanderson K, Johansson G, Lindholm C, Lundberg I (2010). Low level of adjustment latitude--a risk factor for sickness absence. Eur J Pub Health.

[9] Hultin H, Hallqvist J, Alexanderson K, Johansson G, Lindholm C, Lundberg I (2013). Lack of adjustment latitude at work as a trigger of taking sick leave-a Swedish case-crossover study. PLoS One.

[10] Hultin H, Möller J, Alexanderson K, Johansson G, Lindholm C, Lundberg I (2012). Low workload as a trigger of sick leave: results from a Swedish case-crossover study. J Occup Environ Med.

[11] Shaw WS, Pransky G, Fitzgerald TE (2001). Early prognosis for low back disability: intervention strategies for health care providers. Disabil Rehabil.

[12] Karlqvist L, Gard G (2013). Health-promoting educational interventions: a one-year follow-up study. Scand J Public Health.

[13] Larsson A, Karlqvist L, Westerberg M, Gard G (2012). Identifying work ability promoting factors for home care aides and assistant nurses. BMC Musculoskelet Disord.

[14] Larsson A, Karlqvist L, Westerberg M, Gard G (2013). Perceptions of health and risk management among home care workers in Sweden. Phys Ther Rev.

[15] Gard G, Soderberg S (2004). How can a work rehabilitation process be improved?--a qualitative study from the perspective of social insurance officers. Disabil Rehabil.

[16] Gustafsson K, Lundh G, Svedberg P, Linder J, Alexanderson K, Marklund S (2013). Psychological factors are related to return to work among long-term sickness absentees who have undergone a multidisciplinary medical assessment. J Rehabil Med.

[17] Ropponen A, Svedberg P, Koskenvuo M, Silventoinen K, Kaprio J (2014). Physical work load and psychological stress of daily activities as predictors of disability pension due to musculoskeletal disorders. Scand J Public Health.

[18] Kärkkäinen S, Pitkäniemi J, Silventoinen K, Svedberg P, Huunan-Seppälä A, Koskenvuo K (2013). Disability pension due to musculoskeletal diagnoses: importance of work-related factors in a prospective cohort study of Finnish twins. Scand J Work Environ Health.

[19] Muntaner C, Li Y, Ng E, Benach J, Chung H (2011). Work or place? Assessing the concurrent effects of workplace exploitation and area of recidence economic inequality on individual health. Int J Health Serv.

[20] Kim IH, Khang YH, Cho SI, Chun H, Muntaner C (2011). Gender, professional and non-professional work, and the changing pattern of employment-related inequality in poor self-rated health, 1995-2006 in South Korea. J Prev Med Public Health.

[21] Okechukwu CA, Souza K, Davis KD, de Castro AB (2014). Discrimination, harassment, abuse, and bullying in the workplace: contribution of workplace injustice to occupational health disparities. Am J Ind Med.

[22] Main C, Sullivan M, Melin L (2008). Pain management: practical applications of the biopsychosocial perspective in clinical and occupational settings.

[23] Downie A, Williams CM, Henschke N, Hancock MJ, Ostelo RWJG, de Vet HCW, et al. Red flags to screen for malignancy and fracture in patients with low back pain: systematic review. BMJ. 2013;347 10.1136/bmj.f7095 PMC3898572 24335669

[24] Shaw WS, Pransky G, Winters T, Tveito TH, Larson SM, Roter DL (2009). Does the presence of psychosocial "yellow flags" alter patient-provider communication for work-related, acute low back pain?. J Occup Environ Med.

[25] Shaw WS, van der Windt DA, Main CJ, Loisel P, Linton SJ (2009). Early patient screening and intervention to address individual-level occupational factors ("blue flags") in back disability. J Occup Rehabil.

[26] Gray H, Adefolarin AT, Howe TE (2011). A systematic review of instruments for the assessment of work-related psychosocial factors (blue flags) in individuals with non-specific low back pain. Man Ther.

[27] Macfarlane GJ, Pallewatte N, Paudyal P, Blyth FM, Coggon D, Crombez G (2009). Evaluation of work-related psychosocial factors and regional musculoskeletal pain: results from a EULAR task force. Ann Rheum Dis.

[28] Werner EL, Laerum E, Wormgoor ME, Lindh E, Indahl A (2007). Peer support in an occupational setting preventing LBP-related sick leave. Occup Med (Lond).

[29] Chou R, Qaseem A, Snow V, Casey D, Cross JT, Shekelle P (2007). Diagnosis and treatment of low back pain: a joint clinical practice guideline from the American College of Physicians and the American pain society. Ann Intern Med.

[30] Chou R, Shekelle P (2010). Will this patient develop persistent disabling low back pain?. JAMA.

[31] Franche R-L, Corbière M, Lee H, Breslin FC, Hepburn CG (2007). The readiness for return-to-work (RRTW) scale: development and validation of a self-report staging scale in lost-time claimants with musculoskeletal disorders. J Occup Rehabil.

[32] Shaw WS, van der Windt DA, Main CJ, Loisel P, Linton SJ (2009). Decade of the flags working G. Early patient screening and intervention to address individual-level occupational factors ("blue flags") in back disability. J Occup Rehabil.

[33] Kopec JA, Esdaile JM (1998). Occupational role performance in persons with back pain. Disabil Rehabil.

[34] Marhold C, Linton SJ, Melin L (2002). Identification of obstacles for chronic pain patients to return to work: evaluation of a questionnaire. J Occup Rehabil.

[35] Symonds TL, Burton AK, Tillotson KM, Main CJ (1996). Do attitudes and beliefs influence work loss due to low back trouble?. Occup Med (Lond).

[36] Foster NE, Hill JC, O'Sullivan P, Hancock M. Stratified models of care. Best Pract Res Rheumatol. 2013;649-61. 10.1016/j.berh.2013.10.005 24315146

[37] Hemingway H, Croft P, Perel P, Hayden JA, Abrams K, Timmis A (2013). Prognosis research strategy (PROGRESS) 1: a framework for researching clinical outcomes. BMJ.

[38] Hingorani AD, Windt DA, Riley RD, Abrams K, Moons KG, Steyerberg EW (2013). Prognosis research strategy (PROGRESS) 4: stratified medicine research. BMJ.

[39] Foster NE, Mullis R, Hill JC, Lewis M, Whitehurst DG, Doyle C (2014). Effect of stratified care for low back pain in family practice (IMPaCT back): a prospective population-based sequential comparison. Ann Fam Med.

[40] Karran EL, McAuley JH, Traeger AC, Hillier SL, Grabherr L, Russek LN (2017). Can screening instruments accurately determine poor outcome risk in adults with recent onset low back pain? A systematic review and meta-analysis. BMC Med.

[41] Wannstrom I, Nygren A, Asberg M, Gustavsson JP (2008). Different response alternatives in the assessment of job demands. Int Arch Occup Environ Health.

[42] Wannstrom I, Peterson U, Asberg M, Nygren A, Gustavsson JP (2009). Can scales assessing psychological and social factors at work be used across different occupations?. Work.

[43] Wannstrom I, Peterson U, Asberg M, Nygren A, Gustavsson JP (2009). Psychometric properties of scales in the general Nordic questionnaire for psychological and social factors at work (QPS): confirmatory factor analysis and prediction of certified long-term sickness absence. Scand J Psychol.

[44] Bergstrom G, Bodin L, Bertilsson H, Jensen IB (2007). Risk factors for new episodes of sick leave due to neck or back pain in a working population. A prospective study with an 18-month and a three-year follow-up. Occup Environ Med.

[45] Eriksen W (2003). Service sector and perceived social support at work in Norwegian nurses' aides. Int Arch Occup Environ Health.

[46] Finne LB, Knardahl S, Lau B (2011). Workplace bullying and mental distress - a prospective study of Norwegian employees. Scand J Work Environ Health.

[47] Testad I, Mikkelsen A, Ballard C, Aarsland D (2010). Health and well-being in care staff and their relations to organizational and psychosocial factors, care staff and resident factors in nursing homes. Int J Geriatr Psychiatry.

[48] McGettigan P, McKendree J. Interprofessional training for final year healthcare students: a mixed methods evaluation of the impact on ward staff and students of a two-week placement and of factors affecting sustainability. BMC Med Educ. 2015;15 10.1186/s12909-015-0436-9 PMC4623915 26502724

[49] Dallner M EA-L, Gamberale F, Hottinen V, Knardahl S, Lindström K, Skogstad A, Örhede E. Validation of the general Nordic questionnaire (QPSNordic) for psychological and social factors at work. 2000. https://snd.gu.se/sv/catalogue/file/3228. Accessed 1 June 2016.

[50] Linton SJ, Shaw WS (2011). Impact of psychological factors in the experience of pain. Phys Ther.

[51] Biggio G, Cortese CG. Well-being in the workplace through interaction between individual characteristics and organizational context. Int J Qual Stud Health Well-being. 2013;8 10.3402/qhw.v8i0.19823 PMC3576478 23422265

[52] Lakke SE, Soer R, Takken T, Reneman MF (2009). Risk and prognostic factors for non-specific musculoskeletal pain: a synthesis of evidence from systematic reviews classified into ICF dimensions. Pain.

[53] Gard G. Focus on Psychological Factors and Body Awareness in Multimodal Musculoskeletal Pain Rehabilitation. Gard G.. Chapter published in Ed.Bettany-Saltikov and Paz-Lourido “ Physical Therapy Perspectives in the 21st Century - Challenges and Possibilities”,2014,2014.

[54] Le Feuvre N, Kuehni M, Rosende M, Schoeni C (2015). Gendered variations in the experience of ageing at work in Switzerland. Equality Divers Inclusion.

[55] Trnovcova D (2015). Quality of professional and private life during the productive age of employees. Era of science diplomacy: implications for economics, business, management and related disciplines (Edamba 2015).

[56] Lippel K, Vezina M, Bourbonnais R, Funes A (2016). Workplace psychological harassment: gendered exposures and implications for policy. Int J Law Psychiatry.

[57] Linton SJ, Nicholas M, MacDonald S (2011). Development of a short form of the Orebro musculoskeletal pain screening questionnaire. Spine (Phila Pa 1976).

[58] Mehta S, Macdermid JC, Carlesso LC, McPhee C (2010). Concurrent validation of the DASH and the QuickDASH in comparison to neck-specific scales in patients with neck pain. Spine (Phila Pa 1976).

[59] Bremander AB, Petersson IF, Roos EM (2003). Validation of the rheumatoid and arthritis outcome score (RAOS) for the lower extremity. Health Qual Life Outcomes.

[60] Gustafsson U, Grahn B (2008). Validation of the general motor function assessment scale - an instrument for the elderly. Disabil Rehabil.

[61] Josefsson KA, Ekdahl C, Jakobsson U, Gard G (2013). Swedish version of the multi dimensional health assessment questionnaire -- translation and psychometric evaluation. BMC Musculoskelet Disord.

[62] Rubio DM, Berg-Weger M, Tebb SS, Lee ES, Rauch S (2003). Objectifying content validity: conducting a content validity study in social work research. Soc Work Res.

[63] Polit DF, Beck CT (2006). The content validity index: are you sure you know what's being reported? Critique and recommendations. Res Nurs Health.

[64] Tabachnick BG, Fidell LS (2013). Using multivariate statistics.

[65] Altman DG (1991). Practical statistics for medical research.

[66] Chan YH (2003). Biostatistics 104: correlational analysis. Singap Med J.

[67] Tavakol M, Dennick R (2011). Making sense of Cronbach's alpha. Int J Med Educ.

[68] Adamson KA, Prion S (2013). Reliability: measuring internal consistency using Cronbach's alpha. Clin Simul Nurs.

[69] Hirigoyen MF (2016). Bullying as a symptom of the modern world. Ann Med Psychol (Paris).

[70] Gillen PA, Sinclair M, Kernohan WG, Begley CM, Luyben AG. Interventions for prevention of bullying in the workplace. Cochrane Database Syst Rev. 2017;1 10.1002/14651858.CD009778.pub2 PMC6464940 28134445

[71] Nesje K (2017). Professional commitment: does it buffer or intensify job demands?. Scand J Psychol.

[72] Van den Broeck A, Vander Elst T, Baillien E, Sercu M, Schouteden M, De Witte H (2017). Job demands, job resources, burnout, work engagement, and their relationships: an analysis across sectors. J Occup Environ Med.

[73] Bruyere O, Demoulin M, Beaudart C, Hill JC, Maquet D, Genevay S (2014). Validity and reliability of the French version of the STarT back screening tool for patients with low back pain. Spine (Phila Pa 1976).

[74] Bolejko A, Wann-Hansson C, Zackrisson S, Brodersen J, Hagell P (2013). Adaptation to Swedish and further development of the 'Consequences of screening - breast Cancer' questionnaire: a multimethod study. Scand J Caring Sci.

